# Determining the phosphorus release curve for Sunphase HT phytase in nursery pig diets

**DOI:** 10.1093/tas/txad140

**Published:** 2023-12-18

**Authors:** Ty H Kim, Katelyn N Gaffield, Mike D Tokach, Joel M DeRouchey, Jason C Woodworth, Robert D Goodband, Jordan T Gebhardt, Ying Zhou, Xuerong Song, Xiuyi Wu

**Affiliations:** Department of Animal Sciences and Industry, College of Agriculture, Kansas State University, Manhattan, KS 66506-0201, USA; Department of Animal Sciences and Industry, College of Agriculture, Kansas State University, Manhattan, KS 66506-0201, USA; Department of Animal Sciences and Industry, College of Agriculture, Kansas State University, Manhattan, KS 66506-0201, USA; Department of Animal Sciences and Industry, College of Agriculture, Kansas State University, Manhattan, KS 66506-0201, USA; Department of Animal Sciences and Industry, College of Agriculture, Kansas State University, Manhattan, KS 66506-0201, USA; Department of Animal Sciences and Industry, College of Agriculture, Kansas State University, Manhattan, KS 66506-0201, USA; Department of Diagnostic Medicine/Pathobiology, College of Veterinary Medicine, Kansas State University, Manhattan, KS 66506-0201, USA; Wuhan Sunhy Biology Co., Ltd., Wuhan, P.R. China; Wuhan Sunhy Biology Co., Ltd., Wuhan, P.R. China; Wuhan Sunhy Biology Co., Ltd., Wuhan, P.R. China

**Keywords:** bone ash, growth, nursery pigs, phosphorus release, phytase, plasma inositol

## Abstract

A total of 280 pigs (DNA 241 × 600, initially 10.4 ± 0.24 kg) were used in a 21-d study to determine the available P (**aP**) release curve for Sunphase HT phytase (Wuhan Sunhy Biology Co., Ltd., Wuhan, P.R. China) when fed diets with a high phytate concentration. On day 21 post-weaning, considered day 0 of the study, pigs were blocked by average pen body weight (**BW**) and randomly allotted to 1 of 7 dietary treatments with 5 pigs per pen and 8 pens per treatment. Dietary treatments were derived from a single basal diet, and ingredients including phytase, monocalcium P, limestone, and sand were added to create the treatment diets. Treatments included three diets with increasing (0.11%, 0.19%, and 0.27%) aP from monocalcium P, or four diets with increasing phytase (250, 500, 1,000, or 2,000 phytase unit (**FTU**)/kg) added to the diet formulated to 0.11% aP. All diets were corn–soybean meal–canola meal-based and were formulated to contain 1.24% SID Lys, a 1.10:1 total calcium-to-phosphorus ratio, and a calculated 0.32% phytate P. Prior to the beginning of the study, all pigs were fed a diet containing 0.11% aP from days 18 to 21 post-weaning. At the conclusion of the study, 1 pig, closest to the mean weight of each pen, was euthanized, and the right fibula, 10th rib, and metacarpal were collected to determine bone ash and density. After cleaning, bones were submerged in ultra-purified water under a vacuum for 4 h and then weighed to calculate the density (Archimedes principle). For bone ash, bones were processed using the non-defatted method. From days 0 to 21, increasing aP from monocalcium P increased (linear, *P *≤ 0.014) average daily gain (**ADG**), gain-to-feed (**G:F**), and final BW. Pigs fed increasing phytase had increased (linear, *P* ≤ 0.045) ADG, final BW, and plasma inositol concentration as well as improved (quadratic, *P *= 0.023) G:F. For bone characteristics, pigs fed increasing aP from inorganic P had a linear improvement (*P* ≤ 0.019) in fibula bone ash weight and percentage bone ash, rib bone ash weight and bone density, and all metacarpal bone properties, with a quadratic response (*P *≤ 0.030) for fibula bone density and rib percentage ash. Additionally, pigs fed increasing phytase had increased (*P *< 0.05) bone ash weight, percentage bone ash, and bone density in either a linear or quadratic fashion depending on the bone analyzed. The available P release curve generated for Sunphase HT phytase for percentage bone ash combining values from the right fibula, 10th rib, and metacarpal is aP release, % = (0.360 × FTU) ÷ (2,330.250 + FTU).

## Teaser

Pigs fed with increasing phytase had increased bone ash weight, percentage bone ash, bone density, and plasma inositol.

## Introduction

Most swine diets consist of plant-based ingredients containing phytate, which is a major storage form of P in plant-based ingredients (Ravindran et al., 1994). However, phytate-bound P is largely unavailable for digestion and absorption by swine due to inherently low endogenous levels of the digestive enzyme, phytase (Humer et al., 2015). As a result, most swine diets are formulated with an exogenous microbial phytase, which makes plant-derived dietary P more available for utilization (Selle and Ravindran, 2008). In turn, phytase decreases the need for dietary inclusion of inorganic forms of P, which lowers feed costs, reduces antinutritional properties of phytate, and minimizes environmental impact by reducing P excretion.

Dietary phosphorus concentration has a large impact on the development of bones. As a result, bone ash weight and percentage bone ash are used as indicators of phytase efficacy (Gourley et al., 2018; Wensley et al., 2020; Becker et al., 2021). Similar to a study conducted by Gaffield et al. (2023), the current study used bone ash weight and percentage bone ash measurements from multiple bones (fibula, 10th rib, and metacarpal) as indicators of whole-body mineralization. While more investigation is needed to understand the release of P using phytase as measured using different bones, it is hypothesized that an assessment of phytase release using multiple bones will generate predicted available phosphorus (**aP**) release values that are more accurate.

As the feed industry develops new or next-generation phytase sources, an evaluation of their efficacy is needed to properly formulate swine diets. Sunphase HT (Wuhan Sunhy Biology Co., Ltd.), is a bacterial-derived 6-phytase that originates from *Escherichia coli* and is expressed by Pichia Pastoris yeast. Therefore, the objective of this study was to evaluate the effects of a new phytase source (Sunphase HT) on growth performance and bone properties of 10 to 22-kg nursery pigs fed diets with a high phytate concentration (0.32% phytate) and to develop an aP release curve. This is the first study using bone mineralization to provide a range of aP release values for Sunphase HT phytase in pigs.

## Materials and Methods

The protocol used in this experiment was approved by the Kansas State University Institutional Animal Care and Use Committee (4485.31). The phytase premix was analyzed to determine inclusion rate and was found to contain 11,158,000 phytase unit (**FTU**)/kg. Additionally, monocalcium P and limestone were analyzed for Ca and P concentrations, and these values were used in diet formulation (Table 1). The Ca and P concentrations for corn, soybean meal, and canola meal were based on historical analysis of these ingredients used in our previous phytase studies (Gaffield et al., 2023). All diets contained 7.5% canola meal, and due to its high phytate P concentration, resulted in a basal diet with a calculated 0.32% phytate P (Table 3; NRC, 2012). All diets were formulated to a 1.10:1 total calcium-to-phosphorus (**Ca:P**) ratio and to contain 1.24% standardized ileal digestible (**SID**) Lys with other amino acids set to meet or exceed NRC (2012) requirement estimates as a ratio relative to Lys.

**Table 1.** Analyzed ingredient composition (as-fed basis)^1^

Ingredient	Ca, %	P, %
Limestone	37.75	0.01
Monocalcium P	18.57	23.09

^1^Ingredient samples were pooled and analysis was performed at the Kansas State University Soils Lab, Manhattan. Values represent the means of three samples analyzed in duplicate.

### Diet Manufacturing

A single base diet was manufactured at Hubbard Feeds in Beloit, KS. Dietary treatments were derived from eight, 1-ton pallets of basal diet (Table 2). For each treatment diet, a subset of the basal diet from each of the 8 batches along with treatment-specific ingredients including limestone, monocalcium phosphate, sand, and Sunphase HT phytase were mixed to produce the 7 final experimental diets (Table 3). During bagging, complete diet samples were collected from every fourth bag using a feed probe, pooled, and stored at −20 °C.

**Table 2.** Composition of basal batch (as-fed basis)^1^

Item	
Ingredient, %	
Corn	60.94
Soybean meal	29.93
Canola meal	7.61
Sodium chloride	0.61
l-Lys-HCl	0.30
dl-Met	0.10
l-Thr	0.10
l-Val	0.01
Trace mineral premix	0.15
Vitamin premix	0.25
Total	100
Calculated analysis	
Standardized ileal digestible (SID) amino acids	
Lys, %	1.24
Ile:Lys	64
Leu:Lys	130
Met:Lys	33
Met and Cys:Lys	59
Thr:Lys	63
Trp:Lys	18.7
Val:Lys	71
His:Lys	43
Net energy, kcal/kg	2,414
SID Lys:net energy, g/Mcal	5.14
CP, %	22.6
Ca, %	0.33
P, %	0.41
Available P, %	0.07
Standard total tract digestibility P, %	0.17
Phytate P, %	0.32

^1^The basal batch was used as the major ingredient in each experimental diet.

**Table 3.** Ingredient composition of experimental diets (as-fed basis)^1^

	aP, %			Phytase, FTU/kg^2^			
Item	0.11	0.19	0.27	250	500	1,000	2,000
Ingredient, %							
Basal mix	98.62	98.62	98.62	98.62	98.62	98.62	98.62
Limestone	0.37	0.43	0.49	0.37	0.37	0.37	0.37
Monocalcium P	0.19	0.54	0.89	0.19	0.19	0.19	0.19
Sand^3^	0.82	0.41	0.00	0.82	0.81	0.81	0.80
Phytase^4^	—	—	—	0.0022	0.0045	0.0090	0.0179
Total	100	100	100	100	100	100	100
Calculated analysis							
CP, %	22.3	22.3	22.3	22.3	22.3	22.3	22.3
Ca, %	0.50	0.59	0.67	0.50	0.50	0.50	0.50
P, %	0.45	0.53	0.61	0.45	0.45	0.45	0.45
Phytase, FTU/kg	—	—	—	250	500	1,000	2,000
Ca:P ratio	1.10	1.10	1.10	1.10	1.10	1.10	1.10
Phytate P, %	0.32	0.32	0.32	0.32	0.32	0.32	0.32
Analyzed composition^5^							
Ca, %	0.51	0.57	0.76	0.50	0.47	0.53	0.53
P, %	0.48	0.54	0.65	0.48	0.47	0.47	0.49
Phytase, FTU/kg^6^	—	—	—	220	400	960	1,700
Phytic acid, %^6^	1.15	1.16	1.18	1.12	1.17	1.12	1.17

^1^Diets were fed for 21 d starting at ~10.4 ± 0.24 kg BW.

^2^Sunphase HT, Wuhan Sunhy Biology Co., Ltd., Wuhan, P.R. China.

^3^Sand was used to equalize hand-add batch including the addition of limestone, monocalcium P, and phytase when blended with the basal mix.

^4^Phytase was analyzed and contained 11,158,000 FTU/kg (Wuhan Sunhy Biology Co., Ltd.).

^5^Complete diet samples were taken during bagging of experimental diets from every fourth bag and pooled into one homogenized sample per dietary treatment. Samples were stored at −20°C until they were submitted for analysis of Ca (Kansas State University Soils Lab, Manhattan) and P (Midwest Laboratories, Omaha, NE) using the AOAC official method 985.01 (AOAC, 2006).

^6^One sample of each diet was submitted to Eurofins Nutrition Analysis Center (Des Moines, IA) for complete phytase and phytic acid analysis using the AOAC official method 2000.12 (AOAC, 2000) and the method outlined in Analytical Biochemistry Vol. 77:536 to 539 (1977) for the respective analyses.

### Animals and Housing

The study was conducted at the Kansas State University Swine Teaching and Research Center in Manhattan, KS. Each pen (1.22 × 1.22 m) contained a 4-hole, dry self-feeder, and nipple waterer for ad libitum access to feed and water.

A total of 280 pigs (DNA 241 × 600) were weaned at ~21 d of age. At weaning, pigs were randomly allotted to pens and fed common corn-soybean meal-dried whey-based starter diets formulated to contain 1.40 and 1.35% SID lysine in phases 1 and 2, respectively. On day 18 post-weaning, all pigs were fed the diet containing 0.11% aP for a 3-d period. Then, on day 21 post-weaning, considered day 0 of the study, pigs were blocked by BW (initially 10.4 ± 0.24 kg) and randomly allotted to 1 of 7 dietary treatments. There were 5 pigs per pen (3 barrows and 2 gilts or 2 barrows and 3 gilts) and 8 pens per treatment. Treatments included 3 diets containing increasing aP (0.11, 0.19, and 0.27%) from monocalcium P (with limestone added to maintain a Ca:P ratio of 1.10:1), or 4 diets with increasing phytase (250, 500, 1,000, and 2,000 FTU/kg) added to the diet containing 0.11% aP.

Throughout the experiment, pig and feeder weights were recorded every 7 d to determine average daily gain (**ADG**), average daily feed intake (**ADFI**), and gain-to-feed (**G:F**). At the conclusion of the 21-d study, blood was collected from the jugular vein of 1 pig, closest to the mean weight of each pen, using an Ethylenediaminetetraacetic acid (EDTA) anticoagulant blood collection tube to determine plasma inositol concentrations. Blood samples were centrifuged at 4 °C at 1,500 × *g* for 15 min and plasma was frozen for later analysis of plasma inositol (University of North Dakota). For the development of an internal standard, retained serum samples (10 µL) from the laboratory were mixed with 30 µL of 75% methanol containing 100 ng of myoinositol-1,2,3,4,5,6-d_6_ (Medical Isotopes, Pelham, NH). After vortexing and centrifuging for 10 min at 2,000 × *g*, 10 µL of supernatant was injected into an Liquid chromatography-mass spectrometry (LC-MS) system for quantification.

After blood collection, the same pig in each pen was euthanized via penetrating captive bolt, and the right fibula, 10th rib, and metacarpal were collected, individually placed in plastic bags with permanent identification, and stored at −20 °C. For bone analyses, leftover extraneous soft tissue and cartilage caps were removed from each bone. For bone density, bones were submerged in ultra-purified water under vacuum for 4 h. Bones were then suspended in a vessel of water and weighed. The weights were then used to calculate bone density (Archimedes principle; Williams et al., 2023). For bone ash, bones were processed using the non-defatted method (Wensley et al., 2020). Each bone was dried at 105 °C for 7 d in a drying oven and subsequently ashed at 600 °C for 24 h in a muffle furnace. This method was used to determine total bone ash weight and percentage ash relative to dried bone weight (Wensley et al., 2020).

### Chemical Analysis

Two samples of each diet were submitted for analysis of Ca at the KSU Soils Lab, Manhattan, KS (AOAC 985.01, 2006), and average values were calculated. One sample of each diet was submitted for analysis in triplicate for P at Midwest Laboratories, Omaha, NE (AOAC 985.01, 2006). Additionally, one sample of each diet was submitted for complete phytase and phytic acid analysis (Eurofins Nutrition Analysis Center, Des Moines, IA, USA) using the AOAC official method 2000.12 (AOAC, 2000) and the method outlined in Analytical Biochemistry Vol. 77:536 to 539 (1977) for the respective analyses.

### Statistical Analysis

Data were analyzed as a randomized complete block design with pen as the experimental unit, treatment as a fixed effect, and weight block as a random intercept. The base model was fit using the GLIMMIX procedure of SAS version 9.4 (SAS Institute, Inc., Cary, NC, USA). Linear and quadratic contrasts were constructed within increasing inorganic P or phytase treatments. Results were considered significant with *P*-values ≤ 0.05 and were considered marginally significant with *P*-values ≤ 0.10.

For each pen fed the inorganic P diets, the marginal intake of aP per day was calculated according to the following equation: dietary aP% minus 0.11% (the aP in the basal diet) multiplied by ADFI. Using the marginal aP release as the predictor variable, a standard curve was developed for each of the response criteria. The equation for the standard curve was used to calculate aP release from each pen fed the different phytase dosages (250, 500, 1,000, and 2,000 FTU/kg) based on the observed value for each response criterion. Using the pen ADFI, this value was then converted to a marginal aP%.

A mixed model ANOVA with weight block as the random effect was used to evaluate aP release as a function of the calculated phytase dosage using the GLIMMIX procedure. Formulated phytase levels were used to calculate all release values. Additionally, to evaluate the average aP release generated using data from all three bones, treatment and bone were added as fixed effects, and block and pen were added as random intercepts with pen being included to account for the subsampling associated with measuring multiple bones per pig.

A model was fit to calculate release values using non-linear regression to generate estimated aP release curves based on G:F, bone ash weight, percentage bone ash, and bone density using the following functional form:


$$\begin{array}{l} aP\;\;\;release,\%=\frac{a\times (FTU/kg)}{b+(FTU/kg)} \end{array}$$

The model coefficient a is a horizontal asymptote indicating the maximum release of aP for each response, and the model parameter b represents the vertical asymptote. The model parameters were estimated using the *nls* function from the stat package in R (version 4.2.1 [June 23, 2022]; R Core Team, 2021) using the RStudio environment (Version 2.22.12.0.353; RStudio Team, 2021).

## Results

Analysis for Ca, P, and phytase activity of final diets was similar to diet formulation (Table 3). Phytase activity of complete diets increased across the phytase treatments with analyzed phytase concentrations of 220, 400, 960, and 1,700 FTU/kg for the four treatments compared with calculated values of 250, 500, 1,000, and 2,000 FTU/kg, respectively. Analyzed phytase concentrations were below the calculated target for the 500 and 2,000 FTU/kg treatments. However, calculated values of phytase activity were used to determine aP release values. Additionally, analysis of final diets for phytic acid was similar across treatments at 1.15%, which at 28% P (Selle and Ravindran, 2008), results in 0.32% phytate P similar to diet formulation (Table 2).

From days 0 to 21, increasing aP from monocalcium P increased final BW (linear, *P* = 0.014), ADG (linear, *P* = 0.009), and G:F (linear, *P *= 0.002; Table 4). Pigs fed increasing phytase had increased ADG (linear, *P *= 0.011) and final BW (linear, *P* = 0.007). Furthermore, G:F increased quadratically (*P *= 0.023) in pigs fed increasing phytase.

**Table 4.** Effects of increasing aP from monocalcium P or Sunphase HT phytase on nursery pig growth performance and bone ash values^1^^,^^2^

	aP, %^3^			Phytase, FTU/kg^4^					aP, *P = *		Phytase, *P = *	
Item	0.11	0.19	0.27	250	500	1,000	2,000	SEM	Linear	Quadratic	Linear	Quadratic
BW, kg												
Day 0	10.4	10.5	10.4	10.4	10.4	10.4	10.4	0.24	0.440	0.566	0.941	0.696
Day 21	20.7	21.3	22.0	20.8	21.6	21.5	22.1	0.44	0.014	0.877	0.007	0.487
Days 0 to 21												
ADG, g	490	510	552	482	526	505	545	16.7	0.009	0.589	0.011	0.987
ADFI, g	772	776	818	724	773	743	791	23.5	0.143	0.463	0.250	0.258
G:F, g/kg	634	658	676	666	680	679	689	10.1	0.002	0.820	0.001	0.023
Bone characteristics^5^												
Fibula												
Bone ash, g	0.600	0.734	0.821	0.733	0.707	0.844	0.956	0.038	<0.001	0.615	<0.001	0.231
Bone ash, %	43.1	46.2	47.8	44.5	44.9	47.3	48.3	0.848	<0.001	0.468	<0.001	0.158
Bone density, g/mL	1.15	1.22	1.23	1.19	1.20	1.22	1.23	0.012	<0.001	0.030	<0.001	0.005
Rib												
Bone ash, g	0.753	0.913	1.168	0.945	0.922	1.102	1.318	0.061	<0.001	0.530	<0.001	0.408
Bone ash, %	46.4	50.8	51.0	48.5	48.9	51.2	53.1	0.692	<0.001	0.016	<0.001	0.080
Bone density, g/mL	1.20	1.22	1.24	1.20	1.22	1.26	1.25	0.011	0.019	0.932	<0.001	0.047
Metacarpal												
Bone ash, g	0.872	1.073	1.277	1.053	1.160	1.182	1.359	0.039	<0.001	0.972	<0.001	0.018
Bone ash, %	32.5	34.7	37.7	32.5	34.5	35.7	36.1	0.828	<0.001	0.723	<0.001	0.119
Bone density, g/mL	1.13	1.16	1.18	1.15	1.16	1.17	1.18	0.006	<0.001	0.338	<0.001	0.037
Plasma inositol ng/μL	9.59	7.47	8.36	9.82	9.16	11.19	12.22	1.153	0.447	0.281	0.045	0.881

^1^A total of 280 nursery pigs (DNA 241 × 600, initially 10.4 ± 0.24 kg) were used in a 21-d growth trial with 5 pigs per pen and 8 replications per treatment.

^2^ADG, average daily gain; ADFI, average daily feed intake; G:F, gain-to-feed ratio.

^3^Inorganic P was added to the diet by increasing monocalcium P.

^4^Sunphase HT, Wuhan Sunhy Biology Co., Ltd.

^5^One pig per pen (8 pens per treatment) was euthanized and the right fibula, tenth rib, and metacarpal were collected to determine bone density, bone ash weight, and percentage bone ash. After cleaning, bones were submerged in ultra-purified water under vacuum for 4 h. Weights were then collected, and bone density calculated. For bone ash, bones were placed in a drying oven at 105^o^C for 7 d and then ashed in a muffle furnace at 600^o^C for 24 h.

For bone characteristics, increasing aP from monocalcium P resulted in a linear increase (*P* ≤ 0.019) for fibula bone ash weight and percentage bone ash, 10^th^ rib bone ash weight and bone density, and all metacarpal bone properties (Table 4). For fibula bone density and 10^th^ rib percentage bone ash, increasing aP from monocalcium P resulted in a quadratic increase (*P *≤ 0.030).

Similarly, increasing phytase resulted in a linear increase (*P *< 0.001) for fibula bone ash weight and percentage bone ash, with fibula bone density showing a quadratic response (*P *= 0.005). For rib bone properties, increasing dietary phytase resulted in a linear increase (*P *< 0.001) in 10^th^ rib bone ash weight, with rib bone density exhibiting a quadratic increase (*P *= 0.047) and rib percentage bone ash exhibiting a tendency for a quadratic response (*P *= 0.080). Furthermore, pigs fed increasing phytase had a linear increase (*P* < 0.001) in metacarpal percentage bone ash, with metacarpal bone ash weight and bone density increasing in a quadratic fashion (*P *≤ 0.037). Finally, pigs fed increasing phytase had a linear increase (*P* = 0.045) in plasma inositol concentration (Table 4).

The calculated percentage aP release for Sunphase HT phytase followed the same trends as the means listed above with the calculated percentage aP released varying depending on the growth performance, specific bone, and bone characteristics measured (Table 5).

**Table 5.** Calculated aP release values based on different response criteria^1^

	Phytase, FTU/kg^2^					P =	
Item	250	500	1,000	2,000	SEM^4^	Linear	Quadratic
Performance							
ADG	−0.017	0.102	0.037	0.146	0.0433	0.017	0.945
G:F	0.139	0.195	0.194	0.231	0.0455	0.001	0.018
Bone characteristics^3^							
Fibula							
Bone ash, g	0.098	0.073	0.181	0.252	0.0263	<0.001	0.140
Bone ash, %	0.040	0.053	0.155	0.176	0.0318	<0.001	0.105
Bone density, g/mL	0.079	0.082	0.139	0.137	0.0245	<0.001	0.003
Rib							
Bone ash, g	0.086	0.073	0.147	0.228	0.0202	<0.001	0.272
Bone ash, %	0.047	0.062	0.156	0.225	0.0211	<0.001	0.032
Bone density, g/mL	−0.032	0.074	0.290	0.238	0.0529	<0.001	0.027
Metacarpal							
Bone ash, g	0.078	0.119	0.135	0.197	0.0170	<0.001	0.012
Bone ash, %	0.006	0.067	0.117	0.120	0.0271	0.001	0.081
Bone density, g/mL	0.043	0.082	0.139	0.163	0.0262	<0.001	0.031
Average^4^							
Bone ash, g	0.085	0.091	0.150	0.223	0.0204	<0.001	0.076
Bone ash, %	0.020	0.054	0.130	0.159	0.0528	0.007	0.308
Bone density, g/mL	0.045	0.080	0.171	0.166	0.0268	<0.001	0.004

^1^The marginal intake of available P (aP) per day was calculated for each pen using the equation: dietary aP% minus 0.11% (the aP in the basal diet) multiplied by average daily feed intake. A standard curve was then developed for each response criterion using the marginal aP release as the predictor variable. The equation for the standard curve was used to calculate aP release from each pen fed the different phytase dosages based on the observed value for each response criterion.

^2^Sunphase HT, Wuhan Sunhy Biology Co., Ltd.

^3^One pig per pen (8 pens per treatment) was euthanized and the right fibula, rib, and metacarpal were collected to determine bone density, bone ash weight, and percentage bone ash. After cleaning, bones were submerged in ultra-purified water under vacuum for 4 h. Weights were then collected, and bone density calculated. For bone ash, bones were placed in a drying oven at 105 ^o^C for 7 d and then ashed in a muffle furnace at 600 ^o^C for 24 h.

^4^Average aP release values generated using data from the right fibula, rib, and metacarpal.

**Figure 1.**
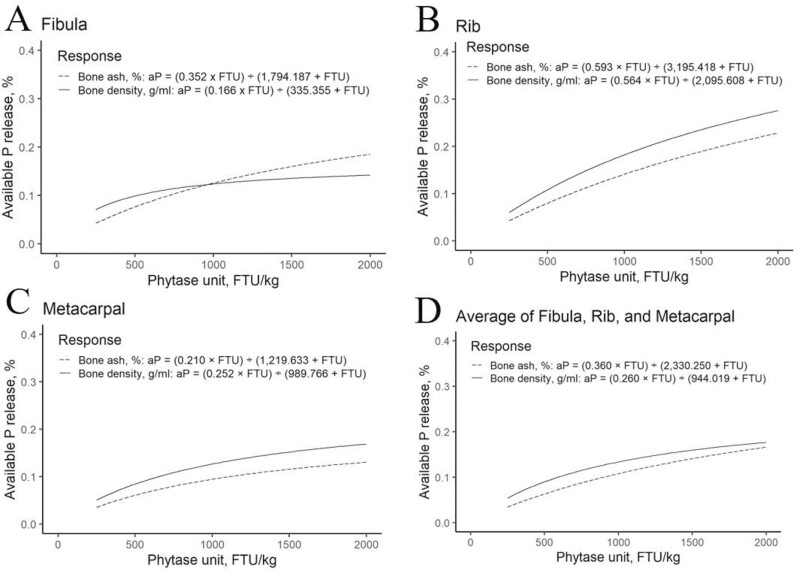
Available P release curves for A) right fibula, B) right rib, C) right metacarpal, and D) average of all three bones including percentage bone ash and bone density generated using the release equations for Sunphase HT from this experiment.


For Sunphase HT, this study has provided an aP release curve for use in swine diets using data from nursery pigs weighing 10 to 21 kg at inclusion levels between 250 and 2,000 FTU/kg (Figure 1). The response criteria considered in this trial influenced the magnitude of aP release differently as FTU inclusion rates increased. An aP release curve was not developed for ADG due to a negative release value at 250 FTU/kg and more erratic values with increasing phytase. However, the aP (%) release equation generated for Sunphase HT for G:F is

$$\begin{array}{l} G:F:\;aP\;release,\%=\frac{0.245\times (FTU/kg)}{175.989+(FTU/kg)} \end{array}$$

The release values for each response criteria averaged across the three bones (fibula, 10th rib, and metacarpal) may provide the most robust estimate of aP release. The aP release equations generated based on measures of bone mineralization are


$$\begin{array}{l} Bone\;ash\;weight:\;aP\;release,\%=\frac{0.344\times (FTU/kg)}{1,156.954+(FTU/kg)} \end{array}$$

$$\begin{array}{l} Percentage\;bone\;ash:aP\;release,\%=\frac{0.360\times (FTU/kg)}{2,330.250+(FTU/kg)} \end{array}$$

$$\begin{array}{l} Bone\;density:\;aP\;release,\%=\frac{0.260\times (FTU/kg)}{944.019+(FTU/kg)} \end{array}$$

## Discussion

The use of phytase in swine diets has become a common practice due to its ability to reduce antinutritional effects and increase nutritional value of feed ingredients (Bedford and Schulze, 1998). The inclusion of phytase in swine diets has shown improvements in production and economics while reducing the impact of the swine industry on the environment (Selle and Ravindran, 2008). Phytases can generally hydrolyze up to 60% to 70% of phytate in the diet, resulting in increased P available to the pig (Newkirk and Classen, 1998; Adeola and Cowieson, 2011). The diets in the study herein contained 1.15% phytic acid, and considering phytic acid is comprised of 28.2% P (Selle and Ravindran, 2008), this provided a calculated value of 0.32% phytate P in each diet.

The first microbial phytases were derived from the fungi *Aspergillus ficuum* in 1990 and commercialized in 1991 (Simons et al., 1990; Dersjant-Li et al., 2014). However, after the initial discovery, there was increased interest in improving the efficacy of phytase sources, as this can be affected by optimal pH, resistance to protease, and affinity to phytate (Dersjant-Li et al., 2014). This led to the discovery and development of *E. coli*-derived bacterial phytase. *Escherichia coli* derived phytases, like Sunphase HT, have been identified as more effective than those derived from fungi (Lei et al., 2013). Specifically, bacterial-derived phytases have been shown to have a greater affinity to phytate and higher resistance to degradation by protease (Adeola and Cowieson, 2011).

Microbial phytase sources can also be classified as 3-phytase or 6-phytase, indicating the position at which hydrolysis of the phytate molecule begins (Selle and Ravindran, 2007). The phytase used in this study, Sunphase HT, is a 6-phytase that originates from *E. coli* and is expressed in Pichia Pastoris yeast. To increase efficacy, this new generation of phytase was developed to be stable up to temperatures of 75 to 80 °C and pH conditions ranging from 4.0 to 5.0.

Phytate is a major storage form of P in cereal grains. To ensure the aP release for Sunphase HT phytase was not limited by available substrate, this study utilized canola meal to increase phytate in the diets (Gaffield et al., 2023). The phytate-bound P concentration of 0.65% for canola meal compares to 0.38% phytate-bound P concentration for soybean meal (NRC, 2012). Newkirk and Classen (1998) observed that 70% to 80% of the phytate in canola meal can be hydrolyzed by phytase. Therefore, the diets used in the current study were formulated to contain 0.32% phytate-bound P. If diets similar to those used in the current study were formulated using corn and soybean meal, the phytate-bound P would be ~0.24%, which is lower than diets containing canola meal. The higher dietary phytate inclusion was utilized to ensure that phytate substrate was not a limiting factor in estimating the phytase response.

Phytic acid is a hexaphosphoric acid ester of myoinositol, or an inositol ring combined with six phosphate molecules, that can inhibit the availability of phosphorus and other minerals (McDowell, 2000). Lu et al. (2019) and Walk et al. (2013) hypothesized that extra-phosphoric effects observed with the use of phytase could be related to the release of myoinositol and mitigation of antinutritional effects of phytate. This could be due to the insulin-like characteristics and function of myoinositol in cellular processes (Croze and Soulage, 2013). Cowieson et al. (2013) observed improved growth performance with the supplementation of myoinositol in broiler chickens. Studies conducted by Cowieson et al. (2017) and Lu et al. (2019) observed increased plasma inositol concentrations when phytase was included up to 3,000 FTU/kg in the diet. The current study observed increased plasma inositol concentrations when phytase was included up to 2,000 FTU/kg, which indicates an increase in the amount of phytate that was completely dephosphorylated.

Phosphorus is the second most abundant mineral in the body and is important for many biological functions (Berndt and Kumar, 2009). The P requirement for maximizing bone mineralization is greater than that to maximize growth performance (Vier et al., 2019). As a result, several studies have determined aP release for different response criteria (Wensley et al., 2020; Becker et al., 2021; Gaffield et al., 2023). Similarly, the current study determined different aP release values for several response criteria. Williams (2023) determined differences in bone mineralization across different bones in nursery pigs. The authors observed fibulas and 2nd ribs are more sensitive to differences in dietary P levels compared to metacarpal and 10^th^ ribs in nursery pigs. However, in a similar study, Williams (2023) observed that 10th ribs were the most sensitive to detect dietary P differences in finishing pigs. As a result, the current study assessed bone properties using multiple bones (fibula, 10th rib, and metacarpal) to have a better understanding of total body mineralization. In the current study, the 10th rib, a non-weight bearing bone, exhibited the highest aP release when assessing bone density and percentage bone ash. However, the metacarpal was observed to have the lowest aP release for percentage bone ash, and the fibula was observed to have the lowest aP release for bone density. As a result of the variation across bones, the aP release for each response criterion averaged across the 3 bones may provide the best information regarding aP release.

When new or next-generation phytase sources are developed and brought to market, an evaluation of their efficacy is needed. The current study observed an improvement in growth performance and bone characteristics in either a linear or quadratic fashion depending on the response. The greatest aP release was observed when Sunphase HT was included at 2,000 FTU/kg for all growth performance criteria as well as bone ash weight and percentage bone ash, indicating phytase levels above our highest inclusion rate tested may release even more aP.

This study has provided a range of aP release values for Sunphase HT phytase in nursery pigs weighing 10 to 21 kg when fed levels between 250 and 2,000 FTU/kg in diets with high phytate concentration. In summary, both growth performance and bone characteristics increased with increasing phytase in the diet. The aP release at different phytase inclusion levels varied depending on response criteria and specific bone. In general, a higher aP release was observed with growth performance compared to bone characteristics, which was not unexpected based on previous research (Wensley et al., 2020; Becker et al., 2021; Gaffield et al., 2023). Equations for aP release were developed for G:F and bone density and percentage bone ash of each of the three bones (fibula, 10th rib, and metacarpal) as well as the average of the three bones. The release values determined using the average of the fibula, 10th rib, and metacarpal may provide the most robust estimate of aP release.
